# Comparison of renal growth in breast fed and artificial fed infants: a cross-sectional study

**DOI:** 10.1186/s13104-023-06368-1

**Published:** 2023-07-10

**Authors:** Aisha Tariq Alam, Iftikhar Ijaz, Muhammad Umer Mukhtar, Muhammad Ahmad Qureshi, Qasim Mehmood, Farnaz Abbas, Khunsa Junaid

**Affiliations:** 1 Department of Pediatrics, King Edward Medical University, Lahore, Pakistan; 2 Medical Student, King Edward Medical University, Nela Gumbad, Hospital Road, 54000 Lahore, Punjab Pakistan; 3 Department of Community Medicine, King Edward Medical University, Lahore, Pakistan

**Keywords:** Breastfeeding, Formula feeding, Kidney growth, Kidney size, Pediatric Nephrology

## Abstract

**Introduction:**

Renal growth in infancy determines renal function in adulthood and can easily be assessed via infant renal volume. Renal growth is influenced by many endogenous and exogenous factors among which nutrition is of prime importance. Worldwide, infants get their nutrition either from breast milk or formula, both of which have controversial roles in kidney growth and development.

**Methods:**

A cross-sectional study was done on healthy infants in the Pediatric Nephrology Department of Mayo Hospital, Lahore. These infants were either breastfed or artificially fed and their kidney volumes were noted to determine any significant difference in kidney size. Both informed and written consent was taken before data collection and the data was analyzed using SPSS version 26.

**Results:**

Out of 80 infants included in our study, 55% were male and 45% were female. The mean age was 8.9 months and the mean weight was 7.6 kg. The mean total kidney volume was 45.38 cm^3^ and the mean relative kidney volume was 6.12 cm^3^/kg. No statistical difference in relative renal volume was found between breastfed and artificially fed infants.

**Conclusion:**

The present study aimed to compare the renal volume and thus renal growth in breastfed versus formula-fed infants. No statistical significance was found in relative renal volume between breastfed and artificially fed infants.

## Introduction

Chronic kidney disease (CKD) is one of the leading global health challenges with a prevalence of 13.4% worldwide. 4.9 to 7.0 million people require renal replacement therapy which is a scarce resource in developing countries. CKD also contributes to a significant financial burden, even in the developed world [1]. Therefore, research on preventive strategies is the need of the hour.

The total number of nephrons in neonates may determine adult kidney health and may portend a predilection to the future development of not only renal but cardiovascular disorders as well [2–4]. The nephron number in neonates depends on various factors like birth weight, prematurity, and gender, with low birth weight being the most significant determining factor [3]. Studies in animal models suggest that rapid kidney growth continues from the prenatal period into early infancy [5]. Whether this period of growth is synonymous with kidney development in humans remains unclear. However, it has been shown that low infant weight gain is associated with lower kidney function in children, supporting the hypothesis that kidney performance is dependent on certain variables prevalent in infancy [6].

Given the potential role that the nephron fund in infants might play in the development of future renal and cardiovascular disorders and the hypothesis that early infancy harbors a period of rapid nephron development, the study of the environmental factors in early infancy that affect kidney development becomes a novel and interesting research question. This idea in itself is not entirely new and various researchers in the past have discussed the potential effects of various interventions in infancy on renal performance [2, 7]. A few studies have also tried to explore the effect of environmental factors that might bring an impact on renal growth in infancy but nothing substantial has been established [6, 8].

While many environmental factors affect renal growth in infants, nutrition is the most important [9]. In Pakistan, as in the rest of the world, the most important source of nutrition for infants is breastfeeding. It has many maternal and infant benefits [9]. In Pakistan, the rate of exclusive breastfeeding is 38% and that of non-exclusive breastfeeding is 67%, which is far from ideal [10]. This study aims to understand the impact of the type of nutrition, either breastfeeding or artificial feeding, on infant kidney function, using renal volume assessed via ultrasound as an indicator of renal growth. In addition, this is the first study ever that reports infant’s kidney sizes in the Pakistani population, an important statistic because malnutrition is rampant in Pakistan which may cause the infant kidney sizes in our population to not conform to international standard ranges.

## Methods and materials

### Setting and time of study

The study was conducted from May 2022 to Sept 2022, in the Division of Pediatric Nephrology, Pediatrics Medical Unit 2, Mayo Hospital, Lahore, Pakistan.

### Study design

Cross-sectional study.

#### Inclusion criteria

All male or female patients of the pediatric population aged 1–12 months, that were either solely breastfed or artificially fed were included as subjects for our study.

#### Exclusion criteria

Children with abnormal renal anatomy which affects renal size were excluded from the study. Such abnormalities included but were not limited to hydronephrosis, reflux, scarring, cysts, congenitally abnormal kidneys, etc. Those who were born pre-term or post-term (before 37 or after 42 weeks) were also excluded from our study.

#### Data collection procedure

Renal volume assessed via ultrasound has been established as a reliable indicator of kidney function. Kidney function itself depends on the functional nephron mass so it’s logical to assess nephron mass as a function of kidney volume in infants, a strategy that we employed in our study [11, 12]. We obtained a detailed history and physical examination of patients presenting to the Department of Pediatrics - Unit 2 at Mayo Hospital, Lahore, Pakistan. Using the WHO standards for child growth, we assessed various anthropometric measures and chose normally nourished infants for this study [13–15].

In our study, the formula-fed infants were fed two types of formulas according to age. Infants less than six months received Formula Feed 1 while infants from age of 6 months to the age of 12 months received Formula Feed 2. The exact contents of the two types of formulas are given below. These two feeds have slight differences in nutritional content according to the physiologic needs of the growing infant.

**Table:** 

Content (per 100 gm of powder)	Formula Feed 1	Formula Feed 2
Energy (Kcal)	487	469
Protein (g)	10.7	14.2
- Whey: Casein	65:35	40:60
Carbohydrates (g)	59.3	59.3
- Sucrose	0	0
Total Fat (g)	23	19.5
- Milk Fat	12	12
- Saturated Fatty Acids	13	9.8
- Monounsaturated Fatty Acid	4.4	3.8
- Polyunsaturated Fatty Acids	2.6	3.4
Sodium (mg)	99	150
Iron (mg)	3	6.2

Both written and informed consent was obtained from the parents. Children were admitted for 10–12 h to the ward from the outpatient department. Chloral hydrate syrup was used at the dose of 50-75 mg/kg for sedation with appropriate management of hypothermia.

Thirty minutes after administration of the syrup, the kidney sizes of 80 children were assessed by a pediatric radiologist with 15 years of experience, using a 3.5 MHz convex US probe. Keeping the child in prone position, three longitudinal and three cross-sectional scans of each kidney were obtained. Kidney volume was calculated in cubic centimeters using the equation for an ellipsoid: volume = mean length x mean width x mean depth x 0.523. Relative kidney volume was calculated as combined kidney volume/body weight (cm^3^/kg) [11].

### Data analysis procedure

Data entered was analyzed using IBM Statistical Packages for Social Sciences 26. Quantitative variables representing age and renal measurements were reported as mean ± SD. Qualitative variables like gender were presented as percentages. As relative kidney volume was s non-normally distributed variable in our study, we employed the Mann-Whitney U test for data analysis. p-value < 0.05 was taken as statistically significant.

## Results

Out of 80 patients that met the inclusion criteria, 55% were male and 45% were female. The mean age was 8.9 months. Our youngest patient was 3 months old and the oldest was 12 months old. The mean weight was 7.6 kg (SD ± 1.7 kg).

Patients in different feeding categories are described in Table 1.

**Table 1:** Feeding Categories

	Breast Fed		Artificially Fed	
	Boys	Girls	Boys	Girls
	27 (33.8%)	13 (16.3%)	17 (21.3%)	23 (28.7%)
Total	40		40	

Renal measurements of patients measured via ultrasound are given in Table 2 as means and standard deviations.

**Table 2:** Renal Measurements

	Right	Left
Length (cm)	5.28 ± 0.7	5.38 ± 0.58
Width (cm)	2.73 ± 0.81	2.82 ± 0.85
Depth (cm)	2.56 ± 1.74	2.42 ± 0.60
Volume (cm^3^)	21.99 ± 6.15	23.39 ± 7.17
Total Kidney Volume (cm^3^)	45.38 ± 12.90	
Relative Kidney Volume (cm^3^/kg)	6.12 ± 1.96	

Male patients (N = 44) had a greater relative kidney volume (6.63 cm^3^/kg ± 2.24) than female patients (N = 36) (5.49 cm^3^/kg ± 1.32).

The results of the statistical analysis are shown in Table 3. Mann-Whitney U test was carried out due to the non-normality of the relative kidney volume variable. The breastfeeding category (N = 40) had a larger mean rank (43.99) than the artificially fed category (N = 40) which had a mean rank of 37.01. A statistically insignificant difference was found (U = 660.5, p = 0.179).

**Table 3:** Statistical Analysis

Relative Kidney Volume	Feeding Type	N	Mean Rank	Sum of Ranks	Mann- Whitney U	p
	Breast Fed	40	43.99	1759.50	660.500	0.179
	Artificially Fed	40	37.01	1480.50		

## Discussion

Renal growth in infants is affected by various endogenous and exogenous factors; among which nutrition is the most important. Renal size however is multifactorial and is governed by many factors like age, sex, BMI, number/stenosis of renal arteries [16]. Most importantly, nutrition governs the growth and size of kidneys in neonates and when not adequately provided can adversely affect kidney function. Calorie and protein deficiency are nutritional deficiencies specifically retarding kidney growth. In Pakistan, where malnutrition is one of the major health problems, nutrition is usually provided to infants by feeding them breast milk or formula milk [17]. Human milk is considered the best source of nutrition for infants as it promotes brain and body growth, modulates intestinal function, strengthens the infant’s immune system, and provides many maternal benefits as well. But it may not be available or suitable in all circumstances. Also, the proteins present in the mother’s milk decrease after the first month postpartum. To overcome this, formula milk has been designed which try to meet the infants’ nutritional requirements as adequately as possible. Commercially produced fortified milk often contains the proteins and nutrients which are required for the optimum growth and development of the infant. Therefore, relying solely on breast milk may lead to inadequate growth of kidneys in infants [18]. But whether human milk or artificial milk is more beneficial for kidney growth in infants is still controversial.

The growth of kidneys in infants is evaluated by measuring renal volume which is the most important parameter in assessing kidney function. It is usually measured via ultrasound, an invariable and reliable method to measure kidney dimensions [17]. Measuring kidney volume in infancy is important as several studies have suggested that poor kidney growth in infants is associated with the development of hypertension and various kidney diseases later in life. It also increases the risk of developing cardiovascular problems in such individuals, which is the major contributor to morbidity and mortality worldwide [6]. In the present study we assessed kidney function by measuring renal volume in infants and tried to compare the size of kidneys in breastfed and formula milk-fed infants.

Of the 80 infants included in our study, 55% were male and 45% were female with a male-to-female ratio of 1.2. The mean age of the infants was 8.9 months and the mean body weight was 7.6 kg. This is in contrast to a study conducted by Schmidt et al [11]. in which the mean body weight of infants was around 3.6 kg.

For the right kidney, the mean dimensions were 5.28 cm, 2.73 cm, and 2.56 cm for length, width, and depth respectively. In comparison, the mean renal dimensions for the left kidney were 5.38 cm, 2.82 cm, and 2.42 cm for length, width, and depth, respectively. The mean renal volume was 21.99 cm^3^ for the right kidney and 23.39 cm^3^ for the left kidney. The mean kidney volume in our study was comparable to that noted by Schmidt et al [11]. Mean total renal volume (mean of the sum of volumes of both kidneys) was 45.38 cm^3^ and relative kidney volume (total kidney volume divided by infant weight) was 6.12 cm^3^/kg. This is also comparable to the readings recorded by Schmidt et al [11] ranging from 6.8 to 7.6 cm^3^/kg in breast-fed and formula-fed infants. In our study, male patients had greater relative kidney volume than female patients.

Due to the non-normality of our data, Mann-Whitney U test was performed. The mean rank was greater in breastfed infants as compared to artificially fed infants. But no statistically significant relationship was found between the feeding type and relative kidney volume in our study participants. Past studies offer conflicting evidence as well. Studies conducted by Miliku et al [6] and Schmidt et al [11] showed that a shorter duration of breastfeeding was related to a smaller combined kidney volume. In another study conducted by Voortman et al [19] longer breastfeeding duration was associated with larger kidney volume and an increased estimated glomerular filtration rate in infants. But in a study conducted by Ece. et al [17], increased renal growth was reported in artificially-fed versus breast-fed healthy infants. Thus, until now, conflicting evidence exists in the medical literature regarding the role of the type of infant nutrition on breastfeeding. Much more medical evidence is needed to conclusively define the role of infant feeding on renal growth. Our study and its findings are a new and unique addition to this growing pool of medical evidence regarding the effect of infant nutrition on kidney growth.

Limitations of our study include its low sample size. Our study was a single-center study. Patients were not followed later on to reassess for continued kidney growth past infancy. Specific age cut-offs for the measurement of kidney size were not used in our study. Renal function markers like creatinine levels were not done because of limited resources. For the future, we believe that a multicenter study, with a large sample size and one that follows kidney growth into well past infancy, will be able to effectively elucidate the effect of breastfeeding/artificial feeding on the kidney function of individuals in infancy and then in adulthood.

## Conclusion

Renal growth in infancy directly affects renal function in adulthood and can be easily assessed by measuring relative kidney volume using ultrasound. The present study aimed to establish a relationship between feeding type and relative kidney volume in infants. No statistically significant relationship was found between relative kidney volume and type of nutrition (either breast milk or formula milk) in infants. Further studies should be conducted to assess the long-term consequences of breastfeeding versus artificial feeding on infant kidney growth and adult kidney function.

## Data Availability

The datasets used and analysed during the current study are available from the author on reasonable request.

## References

[1] Lv JC, Zhang LX, Prevalence, and Disease Burden of Chronic Kidney Disease. Adv Exp Med Biol [Internet]. 2019 [cited 2022 Oct 11];1165:3–15. Available from: https://link.springer.com/chapter/10.1007/978-981-13-8871-2_1. 10.1007/978-981-13-8871-2_1 31399958

[2] Boubred F, Buffat C, Feuerstein JM, Daniel L, Tsimaratos M, Oliver C (2007). Effects of early postnatal hypernutrition on nephron number and long-term renal function and structure in rats. Am J Physiol - Ren Physiol.

[3] Luyckx VA, Ch MBB, Shukha K, Brenner BM (2011). Low Nephron Number and Its Clinical Consequences.

[4] Lackland DT, Bendall HE, Osmond C, Egan BM, Barker DJP (2000). Low birth weights contribute to the high rates of early-onset chronic renal failure in the southeastern United States. Arch Intern Med.

[5] Wu B, Sahoo D, Brooks JD (2013). Comprehensive gene expression changes associated with mouse postnatal kidney development. J Urol.

[6] Children S, Miliku K, Voortman T, Bakker H, Hofman A, Franco OH (2022). Infant breastfeeding and kidney function in school-aged children. Am J Kidney Dis.

[7] Fanos V, Castagnola M, Faa G (2015). Prolonging nephrogenesis in preterm infants: a new approach for prevention of kidney disease in adulthood?. Iran J Kidney Dis.

[8] Escribano J, Luque V, Ferre N, Zaragoza-Jordana M, Grote V, Koletzko B (2011). Increased protein intake augments kidney volume and function in healthy infants. Kidney Int.

[9] Martin CR, Ling PR, Blackburn GL (2016). Review of infant feeding: key features of breast milk and infant formula. Nutrients.

[10] Sabin A, Manzur F, Adil S. Exclusive breastfeeding practices in working women of Pakistan: A cross sectional study. Pakistan J Med Sci [Internet]. 2017 Sep 1 [cited 2022 Oct 11];33(5):1148. Available from: /pmc/articles/PMC5673724/. 10.12669/pjms.335.12827 PMC5673724 29142555

[11] Schmidt IM, Damgaard IN, Boisen KA, Mau C, Chellakooty M, Olgaard K et al. Increased kidney growth in formula-fed versus breast-fed healthy infants. 2004;1137–44. 10.1007/s00467-004-1567-0 15309602

[12] Jovanović D, Gasic B, Pavlovic S, Naumovic R (2013). Correlation of kidney size with kidney function and anthropometric parameters in healthy subjects and patients with chronic kidney diseases. Ren Fail.

[13] Child growth standards [Internet] [cited 2022 Oct 11]. Available from: https://www.who.int/tools/child-growth-standards.

[14] WHO Child Growth Standards. Dev Med Child Neurol [Internet]. 2009 Dec 1 [cited 2022 Oct 11];51(12):1002–1002. Available from: https://onlinelibrary.wiley.com/doi/full/10.1111/j.1469-8749.2009.03503.x.

[15] World Health Organization. WHO child growth standards: length/height-for-age, weight-for-age, weight-for-length, weight-for- height and body mass index-forage: methods and development. World Heal Organ [Internet]. 2006 [cited 2022 Oct 11];1–312. Available from: https://www.who.int/publications/i/item/924154693X.

[16] Glodny B, Unterholzner V, Taferner B, Hofmann KJ, Rehder P, Strasak A et al. Normal kidney size and its influencing factors - a 64-slice MDCT study of 1.040 asymptomatic patients. BMC Urol. 2009;9. 10.1186/1471-2490-9-19 PMC2813848 20030823

[17] Ece A, Gözü A, Bükte Y, Tutanç M, nephrology HK-P. 2007 undefined. The effect of malnutrition on kidney size in children. Springer [Internet]. 2007 Jun [cited 2022 Oct 11];22(6):857–63. Available from: https://link.springer.com/article/10.1007/s00467-006-0338-5. 10.1007/s00467-006-0338-5 17053884

[18] Asim M, Nawaz Y. Child Malnutrition in Pakistan: Evidence from Literature. Child 2018, Vol 5, Page 60 [Internet]. 2018 May 4 [cited 2022 Oct 11];5(5):60. Available from: https://www.mdpi.com/2227-9067/5/5/60/htm. 10.3390/children5050060 PMC5977042 29734703

[19] Voortman T, Bakker H, Sedaghat S, Kiefte–de Jong JC, Hofman A, Jaddoe VWV et al. Protein intake in infancy and kidney size and function at the age of 6 years: The Generation R Study. Pediatr Nephrol [Internet]. 2015 Oct 28 [cited 2022 Oct 11];30(10):1825–33. Available from: https://pubmed.ncbi.nlm.nih.gov/25956700/. 10.1007/s00467-015-3096-4 PMC4549379 25956700

